# School-based gardening, cooking and nutrition intervention increased vegetable intake but did not reduce BMI: Texas sprouts - a cluster randomized controlled trial

**DOI:** 10.1186/s12966-021-01087-x

**Published:** 2021-01-23

**Authors:** Jaimie N. Davis, Adriana Pérez, Fiona M. Asigbee, Matthew J. Landry, Sarvenaz Vandyousefi, Reem Ghaddar, Amy Hoover, Matthew Jeans, Katie Nikah, Brian Fischer, Stephen J. Pont, Daphne Richards, Deanna M. Hoelscher, Alexandra E. Van Den Berg

**Affiliations:** 1 Department of Nutritional Sciences, University of Texas at Austin, 1400 Barbara Jordan Blvd, Austin, TX 78723 USA; 2 Michael & Susan Dell Center for Healthy Living - Department of Biostatistics and Data Science – The University of Texas Health (UTHealth) Science Center at Houston, Austin Campus, Austin, USA; 3 Department of Pediatrics, Dell Medical School, University of Texas at Austin, Austin, TX USA; 4 Texas A&M AgriLife Extension Service, Travis County, Austin, USA; 5 Michael & Susan Dell Center for Healthy Living - Department of Health Promotion and Behavioral Sciences - UTHealth Science Center at Houston, Austin Campus, Austin, USA

**Keywords:** Gardening, Nutrition, Cooking intervention, Hispanic, Low-income, Obesity, Overweight, School-based

## Abstract

**Background:**

Although school garden programs have been shown to improve dietary behaviors, there has not been a cluster-randomized controlled trial (RCT) conducted to examine the effects of school garden programs on obesity or other health outcomes. The goal of this study was to evaluate the effects of a one-year school-based gardening, nutrition, and cooking intervention (called Texas Sprouts) on dietary intake, obesity outcomes, and blood pressure in elementary school children.

**Methods:**

This study was a school-based cluster RCT with 16 elementary schools that were randomly assigned to either the Texas Sprouts intervention (*n* = 8 schools) or to control (delayed intervention, n = 8 schools). The intervention was one school year long (9 months) and consisted of: a) Garden Leadership Committee formation; b) a 0.25-acre outdoor teaching garden; c) 18 student gardening, nutrition, and cooking lessons taught by trained educators throughout the school-year; and d) nine monthly parent lessons. The delayed intervention was implemented the following academic year and received the same protocol as the intervention arm. Child outcomes measured were anthropometrics (i.e., BMI parameters, waist circumference, and body fat percentage via bioelectrical impedance), blood pressure, and dietary intake (i.e., vegetable, fruit, and sugar sweetened beverages) via survey. Data were analyzed with complete cases and with imputations at random. Generalized weighted linear mixed models were used to test the intervention effects and to account for clustering effect of sampling by school.

**Results:**

A total of 3135 children were enrolled in the study (intervention *n* = 1412, 45%). Average age was 9.2 years, 64% Hispanic, 47% male, and 69% eligible for free and reduced lunch. The intervention compared to control resulted in increased vegetable intake (+ 0.48 vs. + 0.04 frequency/day, *p* = 0.02). There were no effects of the intervention compared to control on fruit intake, sugar sweetened beverages, any of the obesity measures or blood pressure.

**Conclusion:**

While this school-based gardening, nutrition, and cooking program did not reduce obesity markers or blood pressure, it did result in increased vegetable intake. It is possible that a longer and more sustained effect of increased vegetable intake is needed to lead to reductions in obesity markers and blood pressure.

**Clinical trials number:**

NCT02668744.

## Background

Studies have consistently shown that increased fruit and vegetable (FV) intake can promote health, lower cardiovascular disease, type 2 diabetes, and some cancers [1–4]. Increased FV may play a role in reduced obesity in adults [5, 6], but the literature supporting this claim in children is unclear [7, 8]. Children in the U.S. do not meet the recommended daily intake for FV, and intake is lowest in low-income and Hispanic populations [9]. Low-income Hispanic families are more likely to live in communities with limited access to healthy foods, often referred to as “food deserts” or “food swamps”, and have less access to quality FV, and increased access to less healthful foods [10, 11]. High levels of acculturation by Hispanics to the dominant U.S. culture might also explain their decreased FV consumption, and increased consumption of unhealthy foods [12, 13]. Further, prices of fresh FV have increased at a much faster rate than high fat/sugar foods [14]. Numerous studies have also shown that lack of FV exposure, preference, knowledge and self-efficacy is linked to lower FV intake in children [15–18]. Evidence-based interventions are needed to improve these psychosocial variables and increase FV access, availability, and intake, particularly in low-income populations.

School gardens have become a common health promotion strategy to increase FV intake in the U.S. In the past two decades, numerous studies have examined the effects of school gardens on psychosocial variables, FV intake, food literacy, and mental health in children [19–24]. Several experimental studies [25, 26] have shown that gardening is linked to lower obesity levels in adults, however few studies have reported on the effects of gardening on obesity and related health measures in children [27]. We completed a pilot study in 2014 in which four schools (~ 400 3rd-5th grade students) were randomly assigned to either a 12-week after-school gardening, nutrition, and cooking intervention (LA Sprouts) or to a control group [27]. Children who participated in LA Sprouts compared to controls experienced significant reductions in BMI z-scores and waist circumference, as well as improved vegetable and fiber intake [27]. To date, LA Sprouts is the only RCT gardening study that has shown promising effects of a garden and nutrition intervention on reducing obesity parameters in children [27]. However, this pilot study was not a cluster-RCT; it was conducted in an after-school setting, and was only 12 weeks long. Assessing gardening, nutrition, and cooking programs using a cluster-RCT in which the program is conducted during school hours for an entire school year is warranted.

Therefore, the overall goal of this project was to conduct a one-year, cluster RCT in elementary schools to examine the effect of a gardening, nutrition, and cooking program, called Texas Sprouts, on 3rd-5th grade children’s dietary intake, obesity outcomes, and blood pressure. We hypothesized that the TX Sprouts intervention compared to the control group would result in the following changes in health outcomes; greater increases in FV intake and greater decreases in sugar sweetened beverage intake, obesity parameters and blood pressure in the intervention children.

## Methods

### Study design and participants

The complete design and methodology of this study has been previously published [28]. This is a school-based cluster RCT in which 16 elementary schools were block randomized by the study biostatistician who was blinded to either: (1) Texas Sprouts Intervention (*n* = 8 schools) or (2) Control (delayed intervention; n = 8 schools). The intervention was implemented in three waves over three years, six schools (three intervention and three control) in wave 1 (2016–2017) and wave 2 (2017–2018) and four schools (two intervention and two control) in wave 3 (2018–2019). All schools had to meet the following inclusion criteria: (1) high proportion of Hispanic children (> 50%); (2) high proportion of children participating in the free and reduced lunch (FRL) program (> 50%), which represents a low-income population; (3) location within 60 miles of central Austin; and (4) no existing garden or gardening program.

All 3rd-5th grade students and parents at the recruited schools were contacted to participate via information tables at “Back to School” and “Meet the Teacher” events, flyers sent home with students, and teachers making class announcements in the fall after the garden had been built at the school. All recruitment materials were available in both English and Spanish. While all 3rd-5th grade students from participating schools received the lessons as part of their in-school curriculum, students/parents had to provide informed written consent to participate in the evaluation measurements. Students and their parents signed assent and consent forms, respectively, approved by the Institutional Review Board (IRB) at UT Austin, as well as the research departments at each of the participating school districts.

### Sample size

The sample size was estimated to test the effects of intervention on child vegetable intake (serving/day), BMI z-scores, and waist circumference, with a power of 80% using a type I error of 0.05, a two-sided test, and assuming equal allocation between the two arms [29, 30]. Sample size estimation, variance (σ^2^) within schools, and the intra-cluster correlation coefficient (ICC) used change data from children who completed our pilot LA Sprouts study [27]. It was estimated that six schools each with 127 children for surveys and measurements were needed to detect the effect size of an increase in 0.5 in vegetable (serving/day) intake, a decrease of 0.065 in BMI z-scores, and a decrease of at least 0.02 cm in waist circumference. Two additional schools per arm were included in case a school decided to withdraw participation. For these reasons, a total sample size of 16 schools was used for this study.

### Intervention

**Fig. 1:**
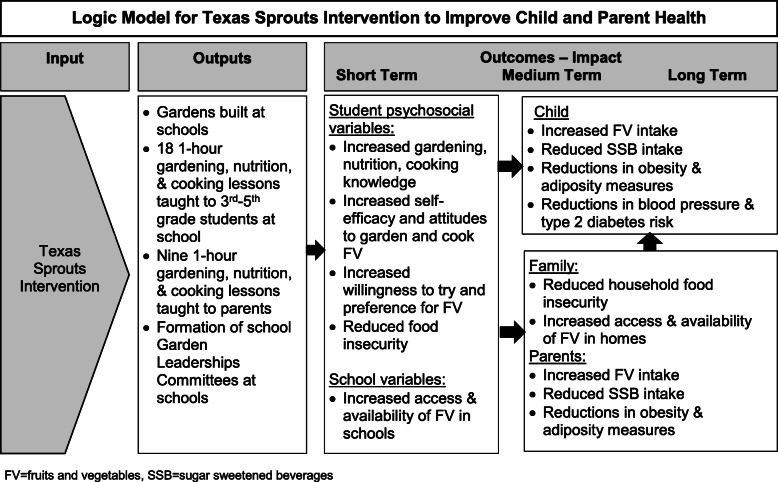
Logic Model for Texas Sprouts Intervention to Improve Child and Parent Health

A social ecological-transactional model was used to shape the Texas Sprouts program, which treats the child as nested within immediate contexts or micro-systems (e.g., school, family, community) that reciprocally interact with each other and the child over time to shape development and behaviors [31, 32]. Texas Sprouts lessons were designed to improve a variety of diet-related psychosocial constructs, including increasing nutrition, gardening, and cooking knowledge, self-efficacy and attitudes, and a child’s willingness to try and preference for FV, and reducingP food insecurity, all of which would lead to increased FV intake and subsequent reductions in obesity parameters and blood pressure. While this analysis focuses on the effects of the intervention compared to control on the main outcomes of FV intake, obesity parameters and blood pressure, we will assess the intervention effects on these diet-related psychosocial variables and how changes in these variables mediate changes in the main outcomes in later analyses. Figure 1 denotes the logic model for this study.

Garden Leadership Committees (GLC) were formed at each intervention school and were comprised of interested stakeholders, such as teachers, parents, community members, school staff, and students. GLCs assisted with the following: a) physical garden design and build; b) hosting several garden workdays; and c) development and implementation of long-term garden maintenance and sustainability plan.

Gardens were built in every intervention school in the spring prior to the academic year of baseline measurements, with the help of the GLC. An average of 150 volunteers attended each garden build. All gardens included: raised vegetable beds; in-ground native and herb beds; a large shed for tools and materials; a whiteboard; and seating for classes. The schools were provided with the materials and supplies needed for garden upkeep (e.g. rakes, hoses, etc.) and for teaching the lessons, (e.g., tables, chairs/benches, cooking grill, portable hand-washing sink, pots/pans, etc.). FV and herbs planted in the garden were chosen based upon seasonality, soil type, and usage of recipes used in the curriculum, but included culturally-specific produce such as tomatoes, squash, peppers, and cilantro.

The Texas Sprouts curriculum was adapted from LA Sprouts [33] and Junior Master Gardener (JMG), a program developed by the Texas A&M AgriLife Extension Service [34]. The following nutrition concepts were included in the final curriculum: (a) healthy cooking/preparation of FV (i.e., low in sugar and fat); (b) making nutritious food choices in different environments; (c) eating locally produced food; (d) low-sugar beverages made with fresh FV; (e) health benefits of FV; f) how to eat healthfully in food desert neighborhoods (neighborhoods lacking easy access to shops selling FV); and (g) food equity and community service. Full-time experienced and trained nutrition and garden educators, who all had college degrees in either horticultural, nutrition, or public health and at least three years of teaching nutrition and gardening lessons to children, taught 18 one-hour Texas Sprouts lessons separately to each 3rd-5th grade class throughout the school year as part of their normal school day. Texas Sprouts curriculum included 18 lessons that were each 60 min in length, whereas the LA Sprouts curriculum only included 12 lessons that were each 90 min in length. However, the content covered in both curricula was essentially identical. Every lesson included either a garden taste-test (seven lessons) or a cooking activity (11 lessons) and a sampling of different “aguas frescas,” which are flavored/infused waters with no added sugar. The student curriculum was designed to be culturally tailored to Hispanics, including culturally appropriate recipes, content, and activities. This curriculum was designed with input from Hispanic families and tested in several pilot studies to ensure that the lessons were culturally sensitive and appropriate [27, 33, 35]. Every lesson was also mapped on Texas Essential Knowledge Standards (TEKS) for science, math, language arts, health, and social studies.

The parents’ curriculum was adapted from the LA Sprouts program [33] and paralleled the nutrition and gardening topics/activities taught to the children. The parent curriculum also included the following topics; importance of family eating, healthy shopping, and increasing home available and access of healthy foods. The parent curriculum was available and taught in both English and Spanish. The garden/nutrition educators taught monthly 60-min Texas Sprouts lessons, for a total of nine lessons, throughout the school year. The dates and times varied widely across school sites, and parent classes were offered in mornings, during school hours, after-school hours, evenings, and even on weekends to account for parent preferences and schedules at the various school sites. Parents were incentivized to attend the lessons with free meals, produce giveaways, groceries, water bottles, t-shirts, garden gloves, raffles for gift cards, and free childcare for children and siblings. The lessons were advertised and promoted by posting flyers, sending home newsletters, and sending out reminder text messages.

### Control

The control schools received a delayed intervention (identical intervention as described above) in the year after the post-testing for that wave. Baseline and post-intervention measurements occurred in the control parents and students within the same time period as the intervention schools. Every control school received a garden, identical in size and structure to the intervention schools. Trained educators taught the 18 school lessons and monthly family lessons at the schools.

### Outcome measurements

Data were collected on children and parents at baseline (within the first month of the beginning of the academic school year) and post-intervention (within the last month of the academic school year). Approximately 10 trained research staff went to each school for a full week to collect all measures at each school. Of note, the Texas Sprouts educator did not participate in the data collection at their respective schools.

Height was measured using a free-standing stadiometer mounted against the wall, to the nearest 0.1 cm (Seca, Birmingham, UK). Waist circumference was measured using NHANES protocol [36]. Weight and bioelectrical impedance were assessed with the Tanita Body Fat Analyzer (model TBF 300). BMI (kg/m^2^) and BMI percentiles were determined using CDC age- and gender-specific values [37]. Blood pressure was measured with an automated monitor with child or adult cuffs (Omron, Schaumberg, IL). Children were asked questions about their age, grade, and sex on a survey. An adapted version of the 2015 School Physical Activity and Nutrition (SPAN) dietary screener was used to assess FV and sugar sweetened beverage consumption in the children [38]. This screener included 14 items, 8-items for vegetables, 2-items for fruit, and 4-items for beverages and each item asked about consumption frequency per day. The items on the screener matched up with what grows in the garden. We assessed the validity of the expanded SPAN questionnaire with a two independent samples (*n* = 70) of children (9–11 y) [39], where vegetable intake reported via questionnaire was compared to that reported via 24-h dietary recalls. Agreement correlations for vegetable items ranged from 0.35 to 0.71 with percentage agreements ranging from 57 to 87%, which suggested moderate to strong agreement [40]. We assessed reliability of this adapted SPAN screener with a separate independent sample (*n* = 76) of children (9–11 y). Test-retest percent agreement was between 71 and 84% for all seven items, which is considered to be moderately reproducible [41]. The final questionnaire packet also included questions on food and meal choice behaviors [42], self-efficacy to cook and/or prepare FV and gardening [33, 43], willingness to try and preferences for FV [44, 45], cooking and gardening attitudes [33], nutrition and gardening knowledge [33], and child food security [46].

Parents were asked to complete a questionnaire packet, which was either given to them at back-to-school or meet-the-teacher nights or brought home to them in their child’s backpack. For this paper, the only parent data that were used was the baseline information regarding their child’s race/ethnicity, and participation in the free and reduced lunch program. However, the parent questionnaire packet included questions on family eating activities [45], household food insecurity, parental dietary intake [47], and home availability of FV [45].

### Dose, reach, and Fidelity of intervention

Texas Sprouts educators completed brief process logs after each child and parent lesson that included: attendance, classes taught outside, number of deviations from planned class activities, number of behavioral disturbances, classroom teacher involvement and student perception/feedback of the lesson’s recipe and taste test of aguas frescas. For “dose”, the number of activities performed within each class was divided by the number of activities scheduled. For “reach”, the number of all children in class and parents with consent attending all the classes was divided by the number of participating children and parents. In addition, an observation survey was developed to assess the educators’ implementation of the lesson. Key study personnel observed each Texas Sprouts educator at least twice per year and completed this observation survey. Both the process logs and observation surveys permitted constructive feedback, additional coaching from key study personnel and iterative learning if performance fell below expected level. Research staff also recorded GLC member information, leadership structure, meeting times/places, sustainability workshops offered/attended, fundraising and media garden events, types of produce grown and harvested, produce use and distribution plans, and community resources leveraged.

### Data management

Study data were collected and managed using REDCap (Research Electronic Data Capture) [48]. Statistical Analyses were completed in SAS version 9.4-TSlevel1M6. Following the recommendations by the National Research Council (US) Panel on Handling Missing Data in Clinical Trials [49]. We are reporting the results of the trial using three type of analysis under three different assumptions to handle missing values for the variables of interest: Complete case analysis (CCA), available case analysis (ACA) and multiple imputation under the missing at random assumption (MI). Children who provided complete data for primary outcomes (i.e., height, weight, BMI, BMI Z-Score, BMI percentile, waist circumference, percentage body fat, systolic and diastolic blood pressure, FV intake, and sugar sweetened beverages from the screener) were used for the complete case analysis, and therefore a fixed sample size was used under this assumption. These same variables are reported for the available case analyses but the sample size changed depending on the number of observations missing in the pre-intervention or post-intervention analyses. We assumed that the missing values of the variables of interest were missing at random and the multiple imputation technique using 10 imputations under a multivariate normal model. The variables included in the imputation model were: sex, age, pre and post primary outcomes, percentage of free and reduce lunch pre and post intervention, and the interaction of the child race/ethnicity. A thousand iterations were used for the burning process and then every ten thousand iterations were used to draw one of the 10 imputations from the model. Autocorrelations and time plots were checked for convergence of the imputation process. A type I error level of 0.05 was used to determine statistical significance of all two-sided statistical tests, and final analyses are presented using Rubin’s rules for reporting weighted summary statistics and *p*-values from the 10 multiple imputations [50]. Weighted cluster summary statistics, and frequency distributions were used to describe pre-intervention characteristics and the change between the pre-intervention and the post intervention characteristics between the eight intervention schools and the eight control schools. Selection of children was assumed to be random within the school.

### Data analyses

Generalized weighted linear mixed models (GLMM) [29, 30] with the identity link were used to test differences between the intervention and the control estimates, with the weighted mean and weighted standard deviations with schools as random clusters for continuous variables (Tables 2 and 3), as well as for the mean of the percentage change in categorical variables (Table 4). GLMM under a multinomial distribution under the cumulative logit link was used for multinomial variables to test for the differences between the intervention and the control schools again assuming the schools as random clusters (Table 3). We implemented Sidak’s methods [51] to account for multiple comparisons pairwise t-tests on the differences in the mean percentages between the intervention and control groups (Table 4).

## Results

**Fig. 2:**
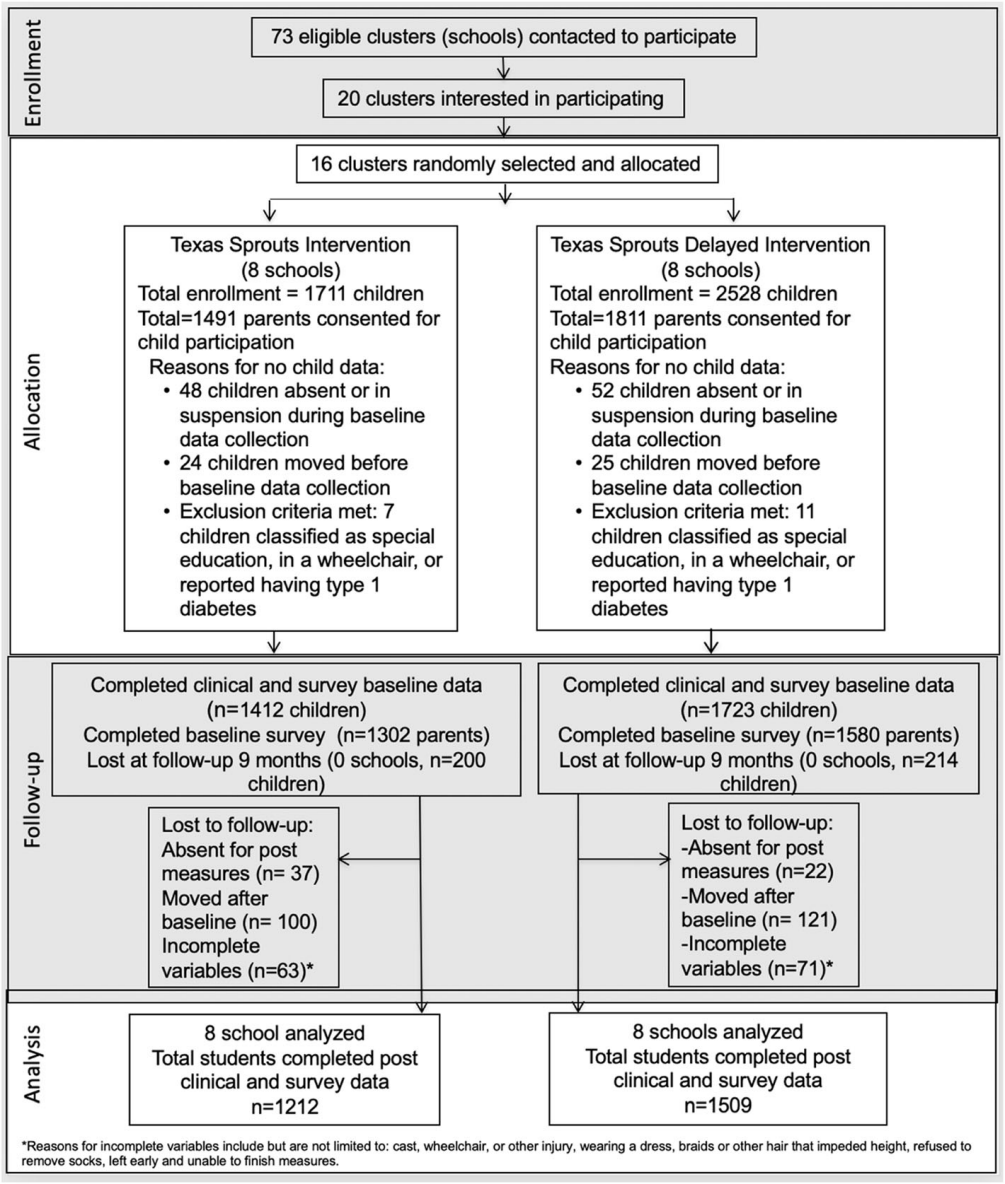
Texas Sprouts Consort Diagram

Figure 2 shows enrollment, randomization and participation in the Texas Sprouts study. Of the 4239 eligible children at the 16 schools, 3302 children (or 78%) consented to be in the study. Of those consented, 3135 children (74% of eligible children or 95% of those consented) completed baseline clinical measures and child surveys and were in the clinical trial. The intervention group included 1412 children (or 45%) and the control group included 1723 children. Approximately 92% (or *n* = 2876, *n* = 1302 parents in intervention and *n* = 1580 parents in control) parents completed baseline surveys. Of the 3135 children who completed baseline clinical and survey measures, 2721 (or 87%) completed post-intervention clinical and survey measures (*n* = 1212 in intervention group and *n* = 1509 in control group).

Complete case and multiple imputation analyses of child baseline demographics of the intervention and control schools are shown in Table 1. The average age of children was 9.2 years and 47% were male. Approximately 64% were Hispanic, and the average percent of children receiving free and reduced breakfast/lunch was 69%. There were no significant differences in any of the demographic variables between intervention and control schools.

**Table 1:** Differences in child demographic, clinical, and dietary characteristics between Texas Sprouts intervention and control groups at baseline

	Total Mean (SE)	Intervention Mean (SE)^a^	Control Mean (SE)^a^	P-value**
Sample size				
CCA	2721	1212	1509	NA
MI	3135	1412	1723	NA
Age (y)				
CCA	9.24 (0.07)	9.28 (0.07)	9.20 (0.07)	0.33
MI	9.23 (0.02)	9.26 (0.06)	9.20 (0.06)	0.47
Male (%) (SE)				
CCA	47.16 (3.88)	46.42 (4.08)	47.89 (3.67)	0.45
MI	47.35 (3.61)	46.99 (3.79)	47.71 (3.44)	0.59
Race/ethnicity % (SE)				
White				
CCA	19.82 (2.75)	21.28 (2.76)	18.37 (2.73)	0.86
MI	19.69 (2.58)	20.90 (2.67)	18.48 (2.51)	0.87
Black				
CCA	8.91 (2.22)	8.61 (2.23)	9.21 (2.20)	0.91
MI	11.10 (2.23)	11.19 (2.32)	11.02 (2.16)	0.85
Hispanic				
CCA	65.80 (3.46)	64.75 (3.46)	66.86 (3.45)	0.92
MI	63.67 (3.25)	62.23 (3.29)	64.42 (3.21)	0.89
Nat.Amer/Asian/Pac.Island/Other				
CCA	5.47 (1.72)	5.37 (1.79)	5.57 (1.65)	0.89
MI	5.88 (1.64)	5.68 (1.69)	6.08 (1.61)	0.75
Eligible FRL %(SE)				
CCA	68.91 (3.55)	66.62 (3.69)	71.21 (3.41)	0.68
MI	68.25 (3.21)	66.47 (3.38)	69.43 (3.07)	0.78

*CCA* complete case analyses; *MI* multiple imputation; *FRL* free and reduced school lunch; *Nat. Amer* Native American; *Pac Island* Pacific Islander

MI analyses is required for all clinical trials and CCA is what is more commonly reported, therefore, both MI and CCA are reported here

^a^ Weighted mean and standard error (SE) of each variable. Weighted refers to the total number of children within each school

**Generalized linear mixed models (GLMM) with linear link was used to compute *p*-values of the continuous variables, GLLM with cumulative logit link was used to compute p-values of the categorical variables

Dose and reach of student and parent classes are reported in Table 2. Texas Sprouts lessons were taught by hired educators during school hours, and make-up days were planned if a lesson was not taught, therefore 100% of the classes were taught to each classroom in grades 3–5. Sometimes classes were shortened (due to testing, assemblies, fire alarms, etc.), but less than 1% of the 18 lessons were modified or shortened across all eight intervention schools. In addition, child lessons were designed to be taught outside, but due to weather issues (i.e., rain, wind, too hot or too cold temperatures), 34% of classes were taught indoors in a classroom. Parent classes were much less attended, despite multiple attempts to offer make-up classes. Only 88.9% of the parent classes were taught, due to no parents showing up for some classes. Only 7.1% of participating parents (*n* = 106) attended one or more Texas Sprouts parent lesson. The average number of parent classes attended was 2.0 ± 1.6, with a range of 1–9. Less than 1% (or *n* = 11) parents attended 50% or more of the offered parent classes (or attended 5 of the 9 classes).

**Table 2:** Dose and reach of Texas Sprouts intervention components

Intervention component	Process Evaluation	Output	Overall (%)
Texas Sprouts student Lesson	Dose delivered	% of classes delivered % of activities taught during class % taught outside	100% 98.5% 66%
	Reach	% of students attending each class	96%
Texas Sprouts Parent Lesson	Dose delivered	% of classes delivered % of activities taught during class	88.9% 100%
	Reach	% of consented parents who attended at least one class % of consented parents who attended ≥50% of classes (5 or more of the 9 offered classes)	7% < 1%

Complete case and multiple imputations analyses of intervention effects on clinical and dietary intake data are shown in Table 3. Using multiple imputation analyses, the intervention compared to control resulted in increased vegetable intake (+ 0.33 vs. + 0.03 freq/d, *p* < 0.01). Similarly, using complete case analyses, the intervention compared to control resulted in increased vegetable intake (+ 0.48 vs. + 0.04 freq/d, *p* = 0.02). The intervention compared to control did not affect fruit or sugar sweetened beverage (SSB) intake. The intervention did not have an effect on any BMI parameters, body fat percentage, waist circumference, or blood pressure. We also analyzed the data using available case analyses, and those results are in the Appendix Table. The results were very similar for the available case analyses. Table 4 shows the changes in BMI status between intervention and control schools. After adjustments were made for multiple comparisons, there was no significant effect of the intervention on BMI status.

**Table 3:** Effects of the Texas Sprouts intervention compared to Control on clinical and dietary outcomes

Outcomes*	Intervention (n = 8 schools) CCA n = 1212 MI n = 1412		Control (n = 8 schools) CCA ***n*** = 1509 MI ***n*** = 1723		Intervention Effect P-value
	Pre	Change	Pre	Change	
	Mean (SE)*	Mean (SE)*	Mean (SE)*	Mean (SE)*	
Height (cm)					
CCA	138.24(0.69)	4.12(0.11)	137.52(0.63)	3.71(0.11)	0.07
MI	138.22(0.64)	4.12(0.11)	137.46(0.59)	3.71(0.10)	0.06
Weight (kg)					
CCA	38.89(0.99)	3.02(0.22)	38.57(0.89)	2.82(0.17)	0.32
MI	38.87(0.91)	3.03(0.21)	38.61(0.84)	2.83(0.16)	0.28
BMI (kg/m^2^)					
CCA	20.03(0.05)	−0.33(0.09)	20.05(0.33)	−0.35(0.08)	0.84
MI	20.03(0.35)	0.33(0.09)	20.08(0.31)	0.35(0.07)	0.87
BMI z-score					
CCA	0.78(0.09)	0.05(0.03)	0.81(0.08)	0.03(0.02)	0.36
MI	0.77(0.09)	−0.04(0.03)	0.81(0.08)	−0.02(0.02)	0.51
BMI Percentile					
CCA	70.11(2.34)	1.20(0.70)	70.98(2.13)	0.60(0.65)	0.39
MI	69.99(2.18)	−0.82(0.70)	71.05(1.99)	−0.39(0.64)	0.53
Waist Circumference (cm)					
CCA	71.34(1.02)	1.10(0.31)	70.62(0.90)	1.50(0.27)	0.31
MI	71.29(0.95)	1.16(0.29)	70.67(0.86)	1.53(0.25)	0.34
Percentage Body Fat					
CCA	25.91(0.73)	−0.33(0.22)	26.09(0.64)	−0.51(0.19)	0.47
MI	25.87(0.68)	−0.34(0.21)	26.10(0.60)	−0.49(0.18)	0.40
Systolic BP (Hg/mm)					
CCA	104.07(0.97)	−0.31(0.93)	102.74(0.86)	0.02(0.89)	0.81
MI	104.10(0.91)	−0.39(0.90)	102.54(0.80)	0.20(0.85)	0.64
Diastolic BP (Hg/mm)					
CCA	68.53(0.81)	−1.20(0.88)	66.21(0.70)	0.22(0.80)	0.28
MI	68.64(0.77)	−1.33(0.84)	66.10(0.65)	0.32(0.76)	0.18
Vegetable intake (freq/d)					
CCA	2.74(0.23)	0.46(0.25)	2.94(0.22)	0.03(0.23)	**0.02**
MI	2.01(0.12)	0.33(0.13)	2.11(0.11)	0.03(0.11)	**0.002**
Fruit intake (freq/d)					
CCA	1.27(0.08)	0.15(0.11)	1.22(0.08)	0.13(0.09)	0.77
MI	1.28(0.08)	0.15(0.10)	1.23(0.07)	0.13(0.09)	0.80
SSB (freq/d)					
CCA	0.58(0.06)	0.01(0.08)	0.61(0.06)	0.09(0.08)	0.15
MI	0.57(0.06)	0.05(0.08)	0.62(0.06)	0.11(0.07)	0.24

*CCA* complete case analyses; *MI* multiple imputation; *BMI* body mass index; *BP* blood pressure; *Freq/d* frequency per day; *SSB* sugar sweetened beverages

*Weighted mean and standard error (SE) of each variable. Weighted refers to the total number of children within each school. Generalized weighted linear mixed models (GLMM) assessed differences between intervention and control groups

**Table 4:** Change in BMI status between intervention and control

Pre-Intervention	Post-Intervention	Intervention (m = 8)	Control (m = 8)	Differences in Mean percentage (SE)^b^	Intervention Effect p-value†	Sidak p-value+
		Mean percentage of students by school (SE)^a^	Mean percentage of students by school (SE)^a^			
Normal/underweight	Normal/underweight	52.64(2.09)	49.34(1.81)	−3.3(2.76)	0.25	0.93
Normal/underweight	Overweight	2.68(0.42)	2.83(0.45)	0.15(0.614)	0.81	1
Normal/underweight	Obese	0.26(0.17)	0.33(0.14)	0.07(0.222)	0.75	1
Overweight	Normal/underweight	3.69(0.55)	5.42(0.45)	1.74(0.712)	0.03	0.23
Overweight	Overweight	11.75(1.15)	13.04(0.95)	1.3(1.49)	0.40	0.99
Overweight	Obese	1.42(0.37)	1.88(0.41)	0.46(0.55)	0.42	0.99
Obese	Normal/underweight	0.21(0.11)	0.06(0.06)	−0.15(0.12)	0.22	0.90
Obese	Overweight	3.08(0.51)	2.60(0.39)	−0.49(0.64)	0.46	1
Obese	Obese	24.28(1.64)	24.50(1.40)	0.22(2.15)	0.92	1

*BMI* body mass index

^a^ Weighted mean percentage and standard error of the mean percentage (SE) of each category change. Weighted refers to total number of participants within each school

^b^Differences of the weighted mean percentage between intervention and control and standard error of the differences in the weighted mean percentage of each category change

**†**T-Tests were used to determine differences in categorical weight status between intervention and control groups by each change of weight status combination

**+**Sidak p-value: adjusted for multiple comparison

## Discussion

Contrary to our hypotheses, the Texas Sprouts intervention, compared to the control group, did not reduce obesity parameters or blood pressure, but resulted in significant increases in vegetable intake. While interventions that increase FV intake have been shown to reduce obesity parameters in adults [6], the results from experimental studies with children have yielded mixed results [52, 53]. Several RCTs with youth have found that nutrition interventions that result in increased vegetable intake have also resulted in reductions in BMI [54] and abdominal adiposity [52], while other studies have shown no effect on obesity levels [53]. Our 12-week afterschool LA Sprouts garden pilot study resulted in improvements in vegetable and fiber intake and reductions in BMI z-scores and waist circumferences [27]. A likely reason that our shorter LA Sprouts program resulted in reductions in obesity parameters, whereas Texas Sprouts did not, is that only two schools received the LA Sprouts program compared to two control schools and the analyses were done on the individual level controlling for school, therefore it was not a cluster analyses. Another possible explanation is that the intensity of the programs varied, in that 12 LA Sprouts lessons, each 90 min in length, were taught weekly for 12 weeks, compared to 18 Texas Sprouts lessons, 60 min in length, that were taught more sporadically throughout the school year. It is also possible that the effects of increasing vegetable intake on reducing obesity parameters requires more intense dissemination initially and/or longer follow-up.

In general, over the past three decades, school-based interventions targeting obesity reductions have yielded mixed results [55–57]. A meta-analysis of 19 school-based RCTs, found that school-based nutrition and/or physical activity interventions were not effective at decreasing BMI compared to control groups [57]. In contrast, another meta-analyses with eight studies showed that school-based nutrition and physical activity intervention did result in reductions in body weight compared to control groups [58]. Research does consistently show that school programs when implemented with complementary community and parent programs can significantly prevent and treat childhood obesity [59, 60]. The available evidence does suggest that no single intervention, in school or elsewhere, is likely to be sufficient to reverse childhood obesity, and that a combined multi-component approach targeting children, parents, the school and the community environment are needed to treat and prevent childhood obesity [61, 62]. In addition, it is important to examine the effects of such multi-component interventions on health benefits beyond obesity parameters, such as metabolic outcomes.

It is also possible that the positive weight loss effects of FV intake are more linked to the displacement of other energy dense foods. In a family-based intervention conducted by Epstein et al. families assigned to a 6-month intervention focused on increasing FV resulted in both increased FV intake and reductions in high fat/sugar foods, and decreased percentage of overweight within the parents, but not child overweight percentages. Whereas families assigned to a 6-month intervention focused solely on decreasing high fat/sugar foods resulted in reductions in high fat/sugar foods, but no effect on changes in FV intake or parental and child overweight percentages [53]. Other interventions have targeted both the increase in FV along with the decrease in high fat and sugary foods and beverages [52, 63]. While the main focus of the Texas Sprouts lessons was to increase FV, we also focused on reducing added sugar, specifically sugar sweetened beverages (SSBs). All 18 Texas Sprouts lessons included a lesson focused entirely on reducing SSBs and every lesson included a tasting of a different agua fresca, or infused water with no added sugar. Contrary to our hypothesis, the intervention did not result in reductions in SSBs. However, the measurement of SSB intake may have been limited given there was only one item measuring this behavior on our survey. Regardless, the increase in vegetable intake, without subsequent displacement of energy dense beverages and/or snacks might explain the null effects of the intervention on obesity measures.

Parent modeling and intake of FV has been consistently shown in the literature to be linked to increased FV intake in the child [64, 65]. While the current study included monthly parent lessons, and was designed to reach both the children at schools and the families at home, parent participation was extremely poor. Only 7% of the participating parents came to one or more parent class. Of those parents that did attend, less than 1% attended 50% of the offered monthly lessons (or 5 of the 9). Despite the incentives (i.e., free babysitting, meals, produce giveaways), reminders, and accommodations (i.e., scheduling classes before, during, and after school and on weekends) that were made to increase parental attendance, participation was poor in every intervention school. The top reasons parents gave for not attending the lessons was lack of transportation and not having enough time. Other school-based interventions have shown success with delivering parental nutrition education through telephone counseling [66], text messaging [67], websites [68], family-fun nights [69], or newsletters/home assignments [43]. However, parental reach in these types of studies is unclear. While there is evidence that parent participation can reduce obesity among children who are overweight or obese in home or clinical settings [70, 71], the evidence is unclear on how parental participation in school based interventions plays a role in obesity prevention in children.

Despite the lack of parental support in the current study, our study did result in in significant and meaningful increases in vegetable intake. In 2017, a review of gardening interventions found that ten of the 14 studies included showed significant improvements in FV intake [23]. Despite the long-term benefits of FV, less than half of the children in the US are consuming the recommended FV intake [9]. Thus, school garden-based interventions offer a promising intervention to effectively increase FV intake in children, which may have a myriad of long-term health benefits beyond the effects on obesity, including reductions in asthma, inflammation, cardiovascular disease, cancer risk, type 2 diabetes risk factors, and improvements in immune response [72–74].

The Whole School, Whole Communities, Whole Child model suggest that health and education must work together whenever possible [75]. Currently, 40 states require nutrition education for all students, yet only 15 states’ explicitly address teacher professional development for health education [76]. This means that most U.S. teachers are mandated to teach nutrition education with little or no training and few curricular resources. Given that schools are one of the most efficient systems for reaching 95% of all U.S. youth and the mandates for schools to teach nutrition education exist, infusing garden and nutrition education into schools offers a practical, meaningful and sustainable way to improve the health of our children.

In addition, the majority of the children in this study receive free and/or reduced breakfast and lunch meals through the National School Lunch Program (NSLP) and the School Breakfast Program (SBP), which means that approximately two thirds of their overall energy intake likely comes from the school. While over the past decade, important policy revisions have been made to the NSLP and SBP, which have resulted in increases FV consumption [77, 78] and increases in healthy eating index scores [79] in school meals, there is still much work to be done to continue to increase the quality of school meals. While the current study exposed children at schools to additional FV during the student lessons, the foodservice department was not involved in the intervention nor was the produce yield from the school garden enough to supplement the school meals in any way. If garden-based interventions incorporated the foodservice more into their programming or grew more produce to supplement the food in the cafeteria, there might even be more improvements in dietary intake among the children.

All Texas Sprouts lessons were taught by well-trained and paid nutrition and gardening educators, in order to control for dosage and reach, and to fully test the effects of the program as designed. However, this does limit the ability to sustain the program moving forward. In the year after the intervention, all 16 schools received a series of garden-based training workshops led by the Sustainable Food Center (SFC), a local non-profit organization that offers school garden trainings, and consultant on this grant. All teachers at that school, not just those that received the Texas Sprouts lessons, were invited to participate in these 2-h workshops, that were either held at the school or at SFC. These workshops covered basic information on gardening, maintenance of physical garden, garden leadership structure and organization, information about local resources/materials, and covered available funding sources. In addition, we secured additional funding from the Sprouts Healthy Communities Foundation to continue to work with these 16 schools to support additional teacher training, at the individual, grade level and whole school level, as well as access to curriculum and resources to continue Texas Sprouts programming. We have recently surveyed 573 school teachers and 173 school administrators on barriers and strategies for sustaining successful school gardening programs, and adequate teacher training, existence of garden leadership committee, and available curriculum were among the top predictors of having a thriving school garden program (paper in review).

There are several other limitations to discuss. The sample was predominately low-income and Hispanic; therefore, results are not generalizable to other race/ethnicities and income levels. However, that was by design, as low-income Hispanic children are at increased risk for obesity. The dietary data was self-reported and the validity and reliability of the adapted SPAN questionnaire only yielded moderate to strong agreement with the 24-h diet recalls and moderate reproducibility. Another limitation is that the intervention is only one school year long, and that follow-up testing may be needed to see long-term effects of increasing vegetable intake on obesity outcomes. In addition, it would have been ideal to add follow-up measures on the control group after receiving the delayed intervention, but this was logistically and financially not possible on a 5-year NIH grant. However, we intend to write additional grants to collect follow-up measures on this cohort. While the current intervention included monthly parent lessons, they were very poorly attended and school-based interventions effectively reaching both the child and parent are warranted. Another limitation is that approximately one third of the garden classes had to be held indoors because of weather, however the full cooking, gardening, and nutrition lesson was still taught indoors. Another limitation is that the current analyses only included the effects of the intervention on the main outcomes (obesity outcomes, blood pressure, and FV and SSB intake), as these variables had the most complete pre/post data and missing at random and multiple imputation (MI) techniques were used for each of these outcomes. In addition, the study was powered on these main outcomes. We did collect other secondary data pre- and post-intervention including: dietary psychosocial variables via survey, academic performance, physical activity via survey and accelerometers, and 24-h dietary recalls, skin carotenoids and fasting blood draws in a subsample of the children. However, as mentioned above, that data is much less complete and/or only collected in a subsample, and the analyses plan would be different for those variables. We intend to examine the effects of the intervention using complete case data on all of those other variables in future analyses.

## Conclusions

This was the first school-based cluster RCT to examine the effects of a school gardening, nutrition, and cooking program on obesity measures and blood pressure. While the intervention did not reduce obesity parameters or blood pressure, it did result in significant and meaningful increases in vegetable intake. The effects of increase vegetable intake on obesity markers might just take longer to reduce obesity markers in pediatric populations. In addition, it important to examine how increased vegetable intake may result in other improvements in health parameters, beyond obesity parameters. We intend to assess the effects of the Texas Sprouts intervention on a variety of metabolic measures obtained from the fasting blood draw, such as glucose, HbA1c, lipids, metabolic syndrome and inflammatory markers. In addition, we intend to assess the effects of the Texas Sprouts intervention on diet-related psychosocial data, energy, macronutrients, healthy eating index, and skin carotenoids, in the near future. We can also examine what behavior changes mediated the increases in vegetable intake and other potential health improvements. School-based garden programs are effective at reaching large numbers of children, fit well into existing school standards and curriculum, provide opportunities for schools to meet nutrition education mandates, and have consistently resulted in increasing vegetable intake in children. There is a need for longer-term follow-up of school-based garden programs. There is also a need to better engage parents in school-based garden programs. Sustaining school garden programs is also challenging. School gardens should be assimilated more into the school culture and curriculum, used by more teachers and students, integrated more into the foodservice and cafeteria, and supported and used more by parents and the community. Research is warranted to help schools succeed at sustaining their school gardening programs.

## Data Availability

The datasets used in the current study are available from the corresponding author on reasonable request.

## Appendix

**Table 5:** Intervention effects on clinical and dietary outcomes using available case analyses

Outcomes^a^		Intervention (m = 8 schools)			Control (m = 8 schools)			Intervention Effect P-value
			Pre	Change		Pre	Change	
	Total n	n	Mean (SE)	Mean (SE)	n	Mean (SE)	Mean (SE)	
Height (cm)	2849	1274	138.20(0.64)	4.11(0.11)	1575	137.46(0.59)	3.71(0.11)	0.07
Weight (kg)	2845	1272	38.85(0.91)	3.03(0.22)	1573	38.61(0.84)	2.84(0.17)	0.32
BMI (kg/m^2^)	2844	1272	20.03(0.35)	−0.33(0.09)	1572	20.08(0.31)	−0.36(0.07)	0.84
BMI z-score	2844	1272	0.77(0.09)	0.05(0.03)	1572	0.81(0.08)	0.03(0.02)	0.42
BMI Percentile	2844	1272	70.01(2.18)	1.13(0.68)	1572	71.04(1.99)	0.58(0.63)	0.42
Waist Circumference (cm)	2840	1271	71.28(0.95)	1.13(0.31)	1569	70.65(0.86)	1.54(0.26)	0.30
Body Fat (%)	2843	1271	25.86(0.68)	−0.35(0.22)	1572	26.09(0.61)	−0.49(0.19)	0.47
Systolic BP (Hg/mm)	2840	1274	104.12(0.91)	−0.30(0.91)	1566	102.54(0.81)	0.13(0.88)	0.76
Diastolic BP (Hg/mm)	2840	1274	68.66(0.78)	−1.25(0.87)	1566	66.10(0.65)	0.26(0.79)	0.25
Vegetable intake (freq/d)	2800	1249	2.76(0.22)	0.47(0.25)	1551	2.95(0.21)	0.01(0.23)	0.01
Fruit intake (freq/d)	2828	1261	1.28(0.08)	0.15(0.10)	1567	1.22(0.07)	0.11(0.09)	0.55
SSB (freq/d)	2833	1266	0.57(0.06)	0.02(0.08)	1567	0.61(0.06)	0.09(0.08)	0.26

*BMI* body mass index; *BP* blood pressure; *freq/d* frequency per day; *SSB* sugar sweetened beverages

^a^Weighted mean and standard error (SE) of each variable. Weighted refers to the total number of children within each school. Generalized weighted linear mixed models (GLMM) assessed differences between intervention and control groups

## Abbreviations

RCT: Randomized controlled trial
FV: Fruits and vegetables
BMI: Body mass index
FTS: Farm to School movement
NSLP: National School Lunch Program
SBP: School Breakfast Program
SSBs: Sugar Sweetened Beverages
CCA: Complete case analysis
ACA: Available case analysis
GLC: Garden Leadership Committees
